# 

**Published:** 1844-04

**Authors:** 


### BOOKS RECEIVED FOR REVIEW.

1. Du Traitement de la Phthisie pulmonaire. Par E. L. Pereyra, M.D.—Bourdeaux, 1843. 8vo, pp. 84.

2. Guide du Médecin Praticien, &c. Par F. L. J. Valleix, M.D., &c.—Paris, 1842-3. 3 vols.

3. Handbuch der Allgemeinen Therapie, &c. Von Dr. Emil Kirchner.—Kiel, 1842. 8vo, pp. 373.

4. Das Medicinische Wien. Von Wilhelm Herzig, M.D., &c.—Wien, 1844. 8vo, pp. 392.

5. Elements of Physiology. By Rudolph Wagner. Part III. On Sensation and Motion, completing the Special Physiology. Translated by R. Willis, M.D.—London, 1844. 10s.

6. Observations on the proximate causes of Insanity. By James Sheppard, Surgeon.—London, 1844. 8vo, pp. 164.

7. The Influence of Climate and other agents on the Human Constitution, with reference to the Causes and Prevention of Diseases among Seamen. By R. Armstrong, M.D., Deputy Inspector of Hospitals and Fleets.—London, 1843. 8vo, pp. 207.

8. Parliamentary Report on the practice of Interment in Towns. By E. Chadwick, Barrister-at-Law.—London, 1843. 8vo, pp. 280.

9. Natural History, Pathology and Treatment of the Epidemic Fever at present prevailing in Edinburgh and other Towns. By J. R. Cormack, M.D.—London, 1843. 8vo, pp. 182. 5s. 6d.

10. The distinction between Instinct and Reason. An Introductory Lecture. By J. Strang, M.D.—London, 1843. 8vo, pp. 44.

11. An Introduction to Practical Organic Chemistry.—London, 1843. 8vo, pp. 90.

12. On the Nature and Treatment of Tic Douloureux, Sciatica and other Neuralgic Disorders. By Henry Hunt, M.D.—London, 1844. 8vo, pp. 192. 6s.

13. Diseases of the Lungs from Mechanical Causes, &c. By G. Calvert Holland, M.D.—London, 1844. 8vo, pp. 100. 4s. 6d.

14. A Letter to the Governors of the Brighton Dispensary, on the Constitution of Dispensaries for the relief of the sick poor. By a Medical Practitioner.—Brighton, 1843. 8vo, pp. 20.

15. Report of the Medical Infirmary Society.—Macao, 1843. 8vo, pp. 62.

16. Anatomical Manipulation; or the Methods of pursuing Practical Investigations in Comparative Anatomy and Physiology. By A. Tulk, and A. Henfrey.—London, 1844. 8vo, pp. 413. 9s.

17. A Treatise on the tonic System of treating affections of the Stomach and Brain. By Henry Scarle, Surgeon.—London, 1843. 8vo, pp. 308. 6s.

18. The Medical Students' Guide and Almanac for 1844.—London, 1844. 8vo, pp. 180. 2s. 6d.

19. A complete condensed practical treatise on Ophthalmic Medicine. By E. O. Hocken, M.D. Part I.—London, 1844. Small 8vo, pp. 108.

20. Elements of Physiology, for the use of Students, and with particular reference to the wants of Practitioners. By Rudolph Wagner, M.D., and Translated from the German with Additions. By Robert Willis, M.D., &c.—London 1844. 8vo, pp. 700.

21. An Experimental and Critical Inquiry into the Nature and Treatment of Wounds of the Intestines. Illustrated by Engravings. By S. D. Gross, M.D., Professor of Surgery in the Louisville Medical Institute. Louisville, (United States,) 1843. 8vo, pp. 220.

22. Minor Surgery, or Hints on the every-day Duties of the Surgeon. By H. H. Smith, M.D. Illustrated by Engravings.—Philadelphia, 1843. 12mo, pp. 303.

23. Practical Manual of the Diseases of the Heart and great Vessels. By J. A. Aran. Translated from the French by W. A. Harris, M.D.—Philadelphia, 1843. Small 8vo, pp. 296.

24. On Regimen and Longevity. By John Bell, M.D.—Philadelphia, 1842. 8vo, pp. 420.

25. Two Essays on the Diseases of the Spine. By R. A. Stafford.—London. 1844. 8vo, pp. 92. 5s.

### INDEX TO VOL. XVII

OF THE

### BRITISH AND FOREIGN MEDICAL REVIEW.

PAGE

Abscess, retro-pharyngeal . 558
Absorption, report on . . 259
Acrodynia . . . 412
Acidulous principle of aliment . 9
Addison, Mr., on blood-corpuscles 92
Age, in relation to diet . . 42
Alcoholic principle of aliment . 10
Alimentary principles . . 7
Aliments, compound . . . 15
Amaurosis, M. Pétrequin on . 155
 asthenic . . . 156
 from lightning . . 157
 traumatic . . ib.
 congestive and irritative . 159
 torpid . . . 163
Amenorrhea . . . 542
Ammonia, carbonate of, in scarlatina 216
Analysis, chemical, Fresenius on . 244
Anatomy, Mr. Paget's report on . 249
Anderson, Dr., his treatment of lunatics . . . 285
Andral, M., on the pathology of the blood . . . 136
Anemia, state of the blood in . 140
Angina pectoris . . . 415
Animal food . . 18
Anuria of old age . . 117
Aphthæ of infants . . 550
Apoplexy, treatment of . . 131
 infantine . . . 553
Army and navy, comparative health of 313
Ascites venosus of old age . 116
Assimilation, report on . . 261
Association, Prov., transactions of . 92
Asthma, spasmodic . . 420

Babington, Mr., on British botany . 248
Ballinghall, Sir G., on military schools 246
Barlow, Mr., on self-control . 245
Bartlett, Dr., on typhus fever . 357
Belladonna in scarlatina . . 222
Belluomini, Dr., on scarlatina . ib.
Bennett, Dr., on the nervous centres 528
Bidder, Dr., on the sympathetic nerve 379
Bile, composition of . . 259
 chemistry of . . . 434
Biliary calculi, chemistry of . . 446
Bird, Dr., his elements of philosophy 523

PAGE

Blood, Andral on the pathology of . 136
 to the head, on determination of 200
 report on the . . 249
 diseased states of . . 480
 on the motion of . . 490
 determination of . . 493
 chemistry of . . 427
 diseased, alleged cause of insanity 526
 in health, state of . . 567
 in inflammation, state of . 570
Bowels, diseases of, in children . 560
Bodenius on scarlatina . . 224
Bone, structure of . . 242
 chemical composition of 261
 chemistry of . . . 444
Books for Review . 299-586
Botany, manual of . . 248
Brain, physiology of . . 265
 diseases of, in army and navy 318-22
 hyperesthesia of . . 418
 tubercle of, in children . . 552
Breathing, capacity of . . 256
Brodie, Sir B., on the duties of students . . . 189
Bronchial phthisis in children . 305
Budge, Dr., on the nerves . . 379
 his experiments . 400
Buffy coat of the blood . . 250
Burder, Dr., obituary of . . 289
Bühlmann, M., on the sputa . . 519

Cæsarian section . . 538
Calcium an element of food . 4
Calculi, renal, chemistry of . 446
Calculous diseases in Mauritius . 288
Cancrum oris and stomatitis . 297
Canstatt, Dr., on the diseases of old age 100
Carbon an element of food . . 2
Carbonic acid, exhalation of . 257
Carpenter, Dr., on physiology . 241
Cataract, Mr. Scott on . . 329
Cerebral hemorrhage in children . 301
Cephalæmatoma . . . 549
Cervix uteri, ulceration of . 546
Chadwick, Mr., his report on interments . . . . 403
Chemistry, pathological treatises on . 424
 Liebig's letters on . . 224

PAGE
Chest, on the statics of . . 152
Children, report on the diseases of . 533
treatise on the diseases of . 293
Chicken-breast, mode of formation 294
Chlorine an element of food . 4
Chossat, M., on inanition . . 344
Chyle, chemistry of . . 427
Circulation, report on the . . 253
capillary, in health . 568
theory of . . . 567
Civiale, M., on the urinary organs . 205
Climate, in relation to diet . . 44
Coagulation of the blood . 249
College of surgeons, new charter of . 279
remarks on . . . 280
Combe, Dr., on the physiology of health 530
Consumption, Dr. Furnivall on . 531
Condiments . . . 22
Convulsions, Dr. Hull on . 203
Cookery, importance of, in dietetics . 34
Corpuscles of the blood . . 250
Craniotomy . . . 538
Croup . . . 556
on tracheotomy in . . 299
Crowfoot, Mr., on fracture of spine . 98
Curling, Mr., on the testis . . 55

Davidson, Dr., his treatise on diet . 1
Deaf and dumb, Dr. Fowler on . 242
Death, different modes of . 500
Diphtheritis of infants . . 555
Dolor cerebralis . . . 418
Delirium tremens, Dr. Watson on . 128
Dietetics, treatises on . . . 1
Diet, rules of . . . . 39
Dietaries, public . . . 47
Digestion, report on . . . 257
Disease, structural, elements of . 498
Drinks, acidulous . . . 21
intoxicating . . . 22
Dropsy, state of the blood in . . 150
Dunglison, Dr. on therapeutics . 242

Eating, times of . . . 45
Echinococcus-hydatid . . 194
Electro-physiology of man . . 246
Elements of diseases, primary . 475
secondary . . . 486
Elsässer, Dr. on the soft occiput . 371
Eclampsia . . . 422
Epidemics of the middle ages . . 522
Epilepsy, Dr. Romberg on . 423
Erysipelas, infantile . . 549
Excito-motor system, anatomy of . 177
Eye, anatomy of . . . 269

Fat, pathological chemistry of . 442
Fæces, chemistry of . 435
Fœtus, diseases of . . 549
Fellows of College of Surgeons, new . 282
Fever, remittent, of children . 563
typhus, Dr. Bartlett's treatise on 357
Fevers, state of the blood in . 143

PAGE
Fevers, in soldiers and sailors . 317-19
Fibro-cellular tissue, report on . . 251
Fistula, vesico-vaginal . 543
Fluorine, an element of food . 4
Food, elements of . . . 2
digestibility of . . 31
cohesive force of . . ib.
chemical constituents of . ib.
proneness of, to acidity . . 33
putrescency . 34
nutritive qualities of . 38
quantity of . . 39
Fowler, Dr. on the deaf and dumb . 242
Fresenius, Dr., on chemical analysis 244
Furnivall, Dr. on consumption . 531

Gangrene in children . . 564
Gelatinous principle of aliment . 11
Glossology, essay on . . 524
Guthrie, Mr. on the urinary organs . 205
Guy, Dr. on medical jurisprudence . 228

Hall, Dr. M. on the reflex function . 171
Hannover, Dr. on the nervous structure 379
Head, on determination of blood to . 200
Headach, Dr. Hull on . . 202
Heat, animal, Jeffreys on . 152
Hereditary diseases, treatise on . 247
Health, Dr. Combe on the preservation of . . . . 530
Heart, its relation to the spinal marrow 404
diseases of, in infants . 560
Hecker, Dr., on the epidemics of the middle ages . . . 522
Hip, contracted . . 420
Hippocrates, Littré's edition of his works 450
Hippocrates, life of . . 459
Hohl, Professor, on Norway prisons 89
Holland, Dr., his statistics of Sheffield 528
Hooping-cough . . 296-559
Hunter, Dr. W., on the gravid uterus 530
Hull, Dr. on determination of blood 200
Hydrocephalus, acute . . 551
chronic . . . 554
Hydrophobia, Dr. Romberg on . 422
Hydatid (Echinococcus) researches on 194
Hydrocele, Mr. Curling on . 66
treatment of . . . 69
Hydrogen, an element of food . 2
Hyperemia, Dr. Williams on . 488
of the brain, in old age . 109
Hypochondria . . . 418
Hysteria, Dr. Watson on . . 134
Dr. Romberg on . . 421

Inanition, experiments on . . 344
Inflammation, state of the blood in 144
Inflammation of nervous centres . 528
Mr. Wharton Jones's report on . 567
first steps of . . . 568
exciting cause of . . 581
Insanity, on the proximate causes of . 526
Instinct and reason, essay on . 525

LIBRARY OF THE
Orleans Parish Medical Society

PAGE
Interments in towns, report on . 403
Iron an element of food . . 4
Irritability an element of disease . 476
Issue, long, in the scalp . 96

Jeffreys, Mr., on the statics of the chest 152
Jones, Mr. Wharton, on inflammation 567
Jurisprudence, medical, Mr. Taylor on 228

Kejestein, its value as a test . . 439
Kennedy, Dr., on scarlatina . 216

Labour, recent literature of . 535
preternatural . . 537-555
Lactation . . . . 274
diseases of . . 541
Lead colic . . . 416
Lee, Dr., his lectures on midwifery . 527
Lehmann, Dr., on physiological chemistry . . . 424
L'Heritier, M., on pathological chemistry . . . ib.
Liebig, his letters on chemistry . 224
Ligneous principle of aliment . 9
Liquid aliments . . . 21
Littré, M., his edition of Hippocrates 450
Livois, M., on the echinococcus . 194
Lunatics, naval, account of . 285
Lungs, structure of . . 256
diseases of, in army and navy 317-20
Lymph, chemistry of . . 427

Magnesium an element of food . 4
Man, physical history of . . 274
Materia medica, Dr. Dunglison on . 242
Medicine, Dr. Williams's principles of 472
Membrane, false, chemistry of . 444
Mercurial sore mouth, treatment of . 125
Mercury, curative effects of . . ib.
Meyer, Dr., on physiology of nerves 379
Measles, report on . . . 561
Medicines, their uses . . 528
Menorrhagia . . . 542
Midwifery, Dr. Lee's lectures on . 527
Dr. West's report on . 533
Military medical schools, on . 246
Milk, chemistry of . . 443
Mucilage an alimentary principle . 8
Muscular tissue, report on . . 252

Natural philosophy, elements of . 253
Navy and army, comparative health of 313
Neligan, Dr., on materia medica . 529
Nerves, minute structure of . . 263
repair of . . . ib.
reflex action of . . ib.
individual, physiology of . 266
hyperesthesia of . . 408
Nervous centres, inflammation of . 528
Nervous system, on the diseases of . 126
system, various treatises on . 379
Newport, Mr., on the reflex function 171
Neuralgia, sciatic . 411

PAGE
Neuralgia, cæliac . . 416
crural . . . 412
cervico-brachial . . ib.
ciliary . . . 411
of the fifth nerve . . 410
Nitrogen, an element of food . 3
Nutrition, report on . . 260
diseases of . . 497

Occiput, the soft, a disease of childhood . . . 371
Oily principle of aliment . . 10
Old age, on the diseases of . . 100
physiology of . . 101
Ophthalmic surgery, treatise on . 233
Orchitis, Mr. Curling on . . 74
Ossification, process of . . 263
Ovaria, dropsical, excision of . 237
diseases of . . 547
Ovum, development of . . 270
Oxygen, an element of food . 3

Paget, Mr., his report on anatomy and physiology . . . 249
Paralysis of infants . . . 555
of serratus magnus . . 99
facial, Dr. Watson on . . 133
Pectinaceous principle of aliment . 9
Pereira, Dr., his treatise on diet . 1
Petersen, M., on the writings of Hippocrates . . . 450
Pétrequin, M., on amaurosis . 155
Pharynx, anatomy of . . 258
Phthisis of infants . . 559
bronchial, in children . 305
pulmonary, in children . 310
mesenteric, in children . 311
Physic, Dr. Watson on the practice of 119
Physiology of animals, Dr. Carpenter on . . . . 241
Mr. Paget's report on 249
Phosphorus, an element of food . 3
Plethora, state of the blood in . 138
Pneumonia senilis . . 112
of infants . . . 559
Polypus uteri . . . 546
of the rectum in infants . . 561
Potassium an element of food . 4
Pregnancy, recent literature of . 533
state of the blood in . . 148
Prisons in Norway, account of . 89
Prognosis, Dr. Watson on . . 124
Proteinaceous principle of aliment . 10
Puberty of girls, age of . 275
Puerperal convulsions . . 538
fever . . . 540
Pus, chemistry of . . 444

Quacks, the fate of . . 193

Regimen, dietetic, in disease . . 51
Reid, Dr., on ventilation . . 532
Remittent fever, Dr. Bartlett on . 357

PAGE
Respiration, Mr. Jeffreys on . 152
report on . . . 254
Respiratory movements . . ib.
Rigor mortis . . 252
Rilliet and Barthez, MM., on the diseases of children . . 293
Rickets in children . . . 566
Ridge, Dr. B., on glossology . 524
Rigby, his edition of Dr. W. Hunter 530
Romberg, Dr., on nervous diseases . 407

Saline principle of aliment . 11
Saliva, secretion of . . 258
Scarlatina, Dr. Kennedy on . 216
report on . . . 562
Schultz, Dr., on the spinal cord . 379
Science, physical sources of . 517
Scrofula in children . . 565
Second editions, pseudo . . 531
Sheffield, statistics of . . 528
Scott, Mr., on cataract . . 329
Scrotum, anatomy of . . 55
on diseases of . . 87
Secretion, diseases of . . 478
Self-control in insanity . . 245
Semen, chemistry of . . 443
Semeiology, Dr. Watson on . 123
Sheppard, Mr., on insanity . 524
Sleepiness, Dr. Hull on . . 201
Smallpox, report on . . 562
Smee, Mr., on physical science . 517
Sodium, an element of food . 4
Sopor hystericus, Dr. Hull on . 202
Spasm of the respiratory muscles 420
Sphincter ani, spasm of . . 420
Spinal cord, anatomy of . 265, 386
physiology of . . 391
Spinal marrow, on the functions of . 171
Spine, fracture of . . 98
Sputa, microscopic characters of . 515
Starch, an alimentary principle . 9
Stilling, Dr. his experiments . . 395
Stilling, Dr., on structure of nerves . 379
Stomach, functions of . . 258
diseases of, in army and navy 318-321
Stomatitis and cancrum oris . . 237
of infants . . . 535
Stricture, Guthrie and Civiale on . 206
treatment of . . 207
Students and practitioners, duties of 189
Sugar, an alimentary principle . 8
Sulphur, an element of food . . 3
Sweat, chemistry of . . 443
Sympathetic system, anatomy of . 381
Sydenham Society . . . 522

Tabes mesenterica . . . 560
Taylor, Mr., on medical jurisprudence 228

PAGE
Teeth, structure of . . 257
Testicle, inflammation of . . 74
irritable . . 83
Testicles, physiology of . . 270
Testis, Mr. Curling's treatise on the . 55
anatomy of . . 57
descent of . . . ib.
undescended, condition of . 59
atrophy of . . 65
Tetanus, Dr. Romberg on . . 421
Therapeutics, Dr. Dunglison on . 242
Tonicity an element of diseases . 476
Tonsils, chronic enlargement of . 558
Trousseau on tracheotomy in croup . 299
Truman, Dr., his treatise on food . 1
Tubercles in the brain of infants . 552
chemistry of . . 445
Tuberculous disease in children . 304

Urinary and genital organs, works on 205
Urine, pathological chemistry of . 437
Urine, incontinence of in children . 561
Uterine hemorrhage . . 538
Uterus, Dr. W. Hunter on the gravid 530
Uterus, occlusion of the mouth of . 536
malposition of . . . ib.
rupture of . . . ib.
Uterus, report on the diseases of . 544
organic diseases of . . 545
Uterus, inflammation of . . ib.
Uterus, extirpation of . . 546

Van Deen, Dr., on the spinal cord . ib.
his experiments . . 393
Varicocele, Dr. Curling on . 85
Vertigo, Dr. Hull on . . 209
Dr. Romberg on . . 413
Ventilation, Dr. Reid on . . 532
Vegetable foods . . 19
Vis nervosa, Dr. M. Hall on . . 172
Vogel, Dr. on animal chemistry . 424
Volkmann, Dr., on the sympathetic nerves . . . 37[illegible]

Wallach, Dr., on structure of nerves 379
Walker, Mr., on ophthalmic surgery 233
Walne, Mr., on excision of ovaria . 237
Wallis, Dr., on the issue in the scalp 96
Warming, Dr. Reid on . . 532
Water an alimentary principle . . 8
Watson, Dr., his practice of physic . 119
West, Dr. his report on midwifery . 533
Williams, Dr., his principles of medicine . . . 472
Women, report on the diseases of . 533

Yellow fever, Dr. Bartlett on . 357

END OF VOL. XVII.

